# Factors associated with the SARS-CoV-2 immunoglobulin-G titer levels in convalescent whole-blood donors: a Chinese cross-sectional study

**DOI:** 10.1038/s41598-024-56462-y

**Published:** 2024-03-13

**Authors:** Donglin Tan, Xinman Du, Jingyun Tang, Humin Liu, Meng Li, Jianxun Kang, Xiaochun Li, Ying Li, Yue Luo, Qing Wang, Xiaobo Gu, Zonghan Zhao, Xuemei Fu, Xue Chen

**Affiliations:** 1 Department of Blood Processing, Chengdu Blood Center, Chengdu, 610041 Sichuan China; 2 Blood Research Laboratory, Chengdu Blood Center, Chengdu, Sichuan China; 3 Department of Blood Testing, Chengdu Blood Center, Chengdu, Sichuan China; 4 Department of Blood Collection, Chengdu Blood Center, Chengdu, Sichuan China

**Keywords:** Health care, Medical research, Risk factors

## Abstract

Blood transfusions from convalescent Severe Acute Respiratory Syndrome Coronavirus 2 (SARS-CoV-2) infected patients could be used to treat patients with severe infections or immunocompromised patients. However, it is necessary to select the optimal donors to maximize the utilization of resources. In this study, we investigated the associations among body mass index (BMI), tobacco smoking, exercise frequency and duration, and alcohol consumption with the SARS-CoV-2 immunoglobulin-G (IgG) antibody titer levels with in the Chinese convalescent blood donor population. Here we show that BMI, smoking habits, and exercise frequency appear to be predictive factors for IgG levels in convalescent male blood donors. However, these variables were not observed as predictive of IgG levels in female convalescent blood donors. The findings could be used to optimize the screening for potential blood donors to treat immunocompromised or severely ill COVID-19 patients.

The coronavirus disease 2019 (COVID-19) pandemic, caused by severe acute respiratory syndrome coronavirus 2 (SARS-CoV-2), remains a threat to global public health^1,2^. Passive immunotherapy with convalescent COVID-19 plasma (CCP) is being employed as a therapeutic procedure for individuals who have been exposed to severe acute respiratory infection by SARS-CoV-2 in many countries^3,4^. The blood of convalescent COVID-19 patients was found to have high levels of neutralizing anti-SARS-CoV-2 antibodies (nAbs). Therefore, CCP could be used as a therapeutic approach for patients with severe COVID-19. Studies have shown that patients treated with CCP in the first few days after hospitalization achieved better prognostic outcomes compared to those who did not^5^.

During the pandemic, the SARS-CoV-2 mutated several times, Which have resulted in a decrease in the effectiveness of vaccines against infection^6^. While small molecule antivirals continue to demonstrate efficacy, it is worth noting that a significant number of monoclonal antibody therapies have encountered challenges in combating the Omicron sublineages due to immune evasion^7^. However, CCP was found to have high levels of antibodies with diverse specificities encompassing all isotypes. Therefore the Association for the Advancement of Blood and Biotherapies (AABB) is now recommending CCP as one of the treatment options for high-risk early-stage COVID-19 outpatients, immunocompromised patients, and individuals with undetectable antibody titers^8,9^.

The neutralizing antibody titer plays a pivotal role in the therapeutic efficacy of using CCP as a treatment for COVID-19^4,10^. Several countries have implemented strategies for CCP screening and have defined high-titer plasma based on diverse serological biomarkers, encompassing the neutralizing antibodies, IgG antibodies, and total antibodies^4,8,11,12^. The Chinese Clinical Treatment Scheme for COVID-19 Convalescent Plasma (Pilot 3rd Edition)^12^ specifies that the qualitative test for COVID-19 serum/plasma IgG antibody yielded a positive result, which remained positive even after dilution by 160 times as per the reagent instructions, or the qualitative test for total antibodies against the novel coronavirus in serum/plasma yielded positive results, which remained unchanged even after a 320-fold dilution as per the reagent instructions. The intervention is applicable to a wide range of patients, including those with common, severe, and critically progressive diseases and high-risk factors.

Previous studies have identified several factors that may affect the antibody response to SARS-CoV-2, including gender, body mass index (BMI), smoking, and exercise habits^13–18^. However, only a few studies were conducted within the Chinese population. Therefore, in this study, we aimed to evaluate the impact of these parameters on antibody titers in Chinese whole-blood donors in the Chengdu region. The findings of this study could be used to identify the several factors that may affect the IgG titers of CCP and thus facilitate the clinical use of plasma to save severe COVID-19 patients.

## Results

The characteristics of the participants associated with SARS-CoV-2 IgG titers are summarized in Table 1. A total of 386 participants were eligible for this study, of whom 120 (31%) had high SARS-CoV-2 IgG titer values. In male donors, the mean BMI of the high IgG titer group was significantly higher than that of the low IgG titer group (*p* = 0.02), the percentage of individuals with a BMI between 0 and 25 in the high IgG titer group was found to be lower at 45%, compared to 60% in the low IgG titer group (*p* = 0.07). Moreover, the proportion of male donors with a BMI of 25 or greater in the high IgG titer group was higher than that in the low IgG titer group (*p* = 0.06). In terms of smoking, the proportion of non-smokers in the high IgG titer group was significantly higher compared to that in the low titer group (*p* = 0.004). Regarding smoking frequency, the high IgG titer group exhibited a significantly lower proportion of smokers who consumed 1–19 cigarettes per week compared to the low titer group (*p* = 0.02). In terms of exercise frequency, the proportion of participants in the high IgG titer group who engaged in physical activity 1–2 times per week was significantly lower compared to those in the low titer group (*p* = 0.05). Conversely, a significantly higher proportion of participants with high IgG titers exercised daily compared to those with low titers (*p* = 0.04). Additionally, we observed a significant increase in the proportion of female donors who donated within 4 weeks after symptom onset in the high titer group compared to the low titer group (*p* = 0.007), as presented in Table 1.

**Table 1:** Participants' characteristics of potential factors according to SARS-CoV-2 IgG titers.

	Male donors				Female donors				Overall donors			
	SARS-CoV-2 IgG titers < 1:160	SARS-CoV-2 IgG titers ≥ 1:160	*p*	*p′*	SARS-CoV-2 IgG titers < 1:160	SARS-CoV-2 IgG titers ≥ 1:160	*p*	*p′*	SARS-CoV-2 IgG titers < 1:160	SARS-CoV-2 IgG titers ≥ 1:160	*p*	*p′*
Number of donors, % (n)	35 (136)	17 (66)	–		34 (130)	14 (54)	–		69 (266)	31 (120)	–	
Age, year, mean ± SD	35.8 ± 9.8	36.5 ± 10.3	0.60		35.7 ± 9.4	37.9 ± 10.5	0.18		35.7 ± 9.6	37.1 ± 10.4	0.20	
Male, % (n)									51 (136)	55 (66)	0.47	
Vaccination times, n, mean ± SD	2.7 ± 0.7	2.9 ± 0.5	0.02		2.8 ± 0.4	2.8 ± 0.4	0.74		2.8 ± 0.6	2.9 ± 0.4	0.08	
Time interval from last vaccination to donation, days, mean ± SD	391 ± 100	420 ± 109	0.10		400 ± 81	420 ± 103	0.24		396 ± 90	420 ± 105	0.04	
Confirmed COVID-19 infection, % (n)	48 (65)	68 (45)	0.005		59 (77)	52 (28)	0.43		53 (142)	61 (73)	0.13	
Occupation, medical personnel, % (n)	1 (1)	0 (0)	0.49		5 (6)	2 (1)	0.42		3 (7)	1 (1)	0.28	
Donated within 4 weeks from symptom onset, % (n)	21 (28)	33 (22)	0.05		11 (14)	26 (14)	0.007		16 (42)	30 (36)	0.001	
BMI, kg/m^2^, mean ± SD	24.5 ± 2.7	25.6 ± 3.8	0.02		22.8 ± 3.0	23.2 ± 3.0	0.59		23.6 ± 3.0	24.5 ± 3.6	0.02	
0 < BMI < 25 kg/m^2^	60 (81)	45 (30)	0.07		80 (104)	80 (43)	0.94		70 (185)	61 (73)	0.13	
BMI > = 25 kg/m^2^	40 (54)	55 (36)	0.06		20 (26)	20 (11)	0.94		30 (80)	39 (47)	0.12	
Missing, % (n)	1 (1)	0 (0)	0.50		0 (0)	0 (0)	–		0.4 (1)	0 (0)	0.52	
Alcohol intake				0.78				0.88				0.67
Never drinking, % (n)	35 (48)	41 (27)	0.45		68 (88)	72 (39)	0.69		51 (136)	55 (66)	0.55	
Quit drinking, % (n)	4 (6)	2 (1)	0.28		3 (4)	2 (1)	0.80		4 (10)	2 (2)	0.30	
Less than or eaual to twice/week, % (n)	47 (64)	50 (33)	0.65		25 (33)	24 (13)	0.98		36 (97)	38 (46)	0.60	
3–4 times/week, % (n)	7 (9)	5 (3)	0.54		2 (3)	0 (0)	0.22		5 (12)	3 (3)	0.30	
Almost every day, % (n)	5 (7)	3 (2)	0.47		1 (1)	0 (0)	0.54		3 (8)	2 (2)	0.41	
Missing, % (n)	1 (2)	0 (0)	0.31		1 (1)	2 (1)	0.61		1 (3)	1 (1)	0.71	
Smoking status				0.02				0.65				0.09
Never smoking, % (n)	40 (55)	62 (41)	0.004		89 (116)	94 (51)	0.24		64 (171)	77 (92)	0.01	
Quit smoking, % (n)	5 (7)	5 (3)	0.82		2 (2)	0 (0)	0.37		3 (9)	3 (3)	0.60	
1–19 cigarettes/day, % (n)	48 (65)	30 (20)	0.02		7 (9)	4 (2)	0.42		28 (74)	18 (22)	0.05	
20 cigarettes or above/day, % (n)	6 (8)	2 (1)	0.14		0 (0)	0 (0)	–		3 (8)	1 (1)	0.16	
Missing, % (n)	1 (1)	2 (1)	0.60		2 (3)	2 (1)	0.70		2 (4)	2 (2)	0.98	
Frequency of sports/exercises				0.04				0.48				0.05
Almost no exercise, % (n)	23 (31)	32 (21)	0.18		40 (52)	39 (21)	0.99		31 (83)	35 (42)	0.45	
1–2 times/week, % (n)	38 (52)	24 (16)	0.05		25 (33)	26 (14)	0.75		32 (85)	25 (30)	0.21	
3–4 times/week, % (n)	22 (30)	15 (10)	0.25		15 (20)	7 (4)	0.12		19 (50)	12 (14)	0.07	
Almost every day, % (n)	16 (22)	29 (19)	0.04		18 (24)	24 (13)	0.58		17 (46)	27 (32)	0.05	
Missing, % (n)	1 (1)	0 (0)	0.50		1 (1)	4 (2)	0.19		1 (2)	2 (2)	0.44	
Time duration of sports/exercises				0.50				0.11				0.19
Less than or equal to 2 h/week, % (n)	67 (91)	62 (41)	0.50		78 (101)	78 (42)	0.72		72 (192)	69 (83)	0.63	
3–4 h/week, % (n)	16 (22)	14 (9)	0.65		6 (8)	0 (0)	0.08		11 (30)	8 (9)	0.28	
5 h or above/week, % (n)	16 (22)	23 (15)	0.26		15 (20)	22 (12)	0.47		16 (42)	23 (27)	0.15	
Missing, % (n)	1 (1)	2 (1)	0.62		1 (1)	0 (0)	0.46		1 (2)	1 (1)	0.99	

*P* for all the variables other than age are age-adjusted. The age-adjusted p-values were estimated by the regression method (linear regression for continuous variables and logistic regression for categorical variables).

*P'* were estimated using Pearson chi-square test (or Fisher's exact test when Pearson chi-square test was not valid), for each multiple categorical variable after excluding missing data.Means ± standard deviation for continuous variables and percentages (numbers of cases) for categorical variables.

The associations between the interest variables and high IgG titer values are summarized in Table 2. In male participants, an increase of 1 kg/m^2^ in the BMI was associated with a higher OR of having high IgG titer values (OR 1.12, 95% CI: 1.00–1.26). Conversely, exercising 1 to 2 times per week (OR 0.35, 95% CI: 0.13–0.91) and smoking (OR 0.30, 95% CI: 0.15–0.63) were associated with a lower OR of having high IgG titer values. No significant association was observed between the IgG titer values and BMI, exercise, and smoking in female participants.

**Table 2:** Odds Ratios (95% CIs) of high-titer CCPs according to potential predictive factors.

	Unadjusted Odds Ratios (95% CIs)	Multivariable Odds Ratios (95% CIs)*	Multivariable Odds Ratios (95% CIs)†	Multivariable Odds Ratios (95% CIs)‡
Overall donors				
Number of high-titer donors/number of donors	120/386			
BMI (per 1 kg/m^2^ increment)	1.09(1.02–1.16)	1.00(1.08–1.01)	1.09(1.01–1.18)	1.09(1.01–1.18)
Overweight (BMI > = 25 kg/m^2^ vs. 0 < BMI < 25 kg/m^2^)	1.49(0.95–2.34)	1.37(0.84–2.23)	1.49(0.90–2.47)	1.45(0.86–2.44)
Alcohol intake				
Never or quit drinking alcohol (Ref.)	1	1	1	1
Currently drinking alcohol	0.94(0.61–1.45)	0.87(0.54–1.40)	0.93(0.56–1.55)	0.95(0.57–1.58)
Smoking status				
Never or quit smoking (Ref.)	1	1	1	1
Currently smoking	0.53(0.31–0.90)	0.41(0.22–0.74)	0.38(0.20–0.70)	0.36(0.19–0.67)
Frequency of sports/exercises				
Almost no exercise (Ref.)	1	1	1	1
1–2 times/week	0.70(0.40–1.22)	0.63(0.35–1.13)	0.56(0.30–1.04)	0.54(0.29–1.02)
More than 3 times/week	0.95(0.57–1.58)	0.94(0.55–1.59)	0.71(0.36–1.43)	0.65(0.32–1.33)
Time duration of sports/exercises				
Less than or equal to 2 h/week (Ref.)	1	1	1	1
3 h or above/week	1.16(0.72–1.86)	1.32(0.80–2.19)	1.41(0.72–2.74)	1.55(0.78–3.07)
Male donors				
Number of high-titer male donors/number of male donors	66/202			
BMI (per 1 kg/m^2^ increment)	1.13(1.02–1.25)	1.11(1.00–1.23)	1.14(1.02–1.27)	1.12(1.00–1.26)
Overweight (BMI > = 25 kg/m^2^ vs. 0 < BMI < 25 kg/m^2^)	1.80(0.99–3.26)	1.63(0.86–3.08)	1.87(0.94–3.71)	1.67(0.82–3.41)
Alcohol intake				
Never or quit drinking alcohol (Ref.)	1	1	1	1
Currently drinking alcohol	0.92(0.50–1.67)	0.81(0.43–1.53)	0.90(0.44–1.81)	0.89(0.44–1.82)
Smoking status				
Never or quit smoking (Ref.)	1	1	1	1
Currently smoking	0.41(0.22–0.75)	0.37(0.19–0.71)	0.32(0.16–0.64)	0.30(0.15–0.63)
Frequency of sports/exercises				
Almost no exercise (Ref.)	1	1	1	1
1–2 times/week	0.45(0.21–1.00)	0.48(0.21–1.09)	0.36(0.14–0.93)	0.35(0.13–0.91)
More than 3 times/week	0.82(0.40–1.69)	0.93(0.44–1.97)	0.61(0.23–1.68)	0.55(0.19–1.55)
Time duration of sports/exercises				
Less than or equal to 2 h/week (Ref.)	1	1	1	1
3 h or above/week	1.21(0.65–2.25)	1.47(0.75–2.88)	1.59(0.65–3.92)	1.89(0.74–4.82)
Female donors				
Number of high-titer female donors/number of female donors	54/184			
BMI (per 1 kg/m^2^ increment)	1.04(0.94–1.16)	1.04(0.93–1.16)	1.04(0.93–1.16)	1.03(0.92–1.16)
Overweight (BMI > = 25 kg/m^2^ vs. 0 < BMI < 25 kg/m^2^ )	1.02(0.47–2.25)	1.00(0.44–2.27)	1.05(0.46–2.42)	0.95(0.39–2.28)
Alcohol intake				
Never or quit drinking alcohol (Ref.)	1	1	1	1
Currently drinking alcohol	0.81(0.39–1.68)	0.88(0.41–1.88)	0.97(0.44–2.15)	0.89(0.39–2.00)
Smoking status				
Never or quit smoking (Ref.)	1	1	1	1
Currently smoking	0.51(0.11–2.46)	0.55(0.11–2.74)	0.52(0.10–2.66)	0.52(0.10–2.79)
Frequency of sports/exercises				
Almost no exercise (Ref.)	1	1	1	1
1–2 times/week	1.05(0.47–2.35)	1.12(0.48–2.62)	1.08(0.46–2.58)	1.13(0.46–2.74)
More than 3 times/week	0.96(0.45–2.04)	0.85(0.39–1.88)	0.81(0.28–2.34)	0.76(0.26–2.25)
Time duration of sports/exercises				
Less than or equal to 2 h/week (Ref.)	1	1	1	1
3 h or above/week	1.03(0.48–2.22)	0.90(0.39–2.05)	1.02(0.33–3.16)	1.09(0.33–3.59)

*Adjusted for age, vaccination times, diagnosis status, and time interval from symptom onset to donation.

^†^Overweight is adjusted further for alcohol intake, smoking status, frequency and time duration of sports/exercises. Other variables were adjusted further for each other variable.

^‡^Adjusted further for time interval from last vaccination to blood donation (quartiles), and the missing data for this variable (n = 83) were included as one group of the categorical variable in the model.

Missing data of variables other than the time interval from last vaccination to blood donation was deleted for corresponding models.

## Discussion

The early identification of patients with high plasma antibody titers against COVID-19 could facilitate the treatment of immune-compromised patients through blood transfusions. Various factors could influence the antibody titer in convalescent COVID-19 patients. In this cross-sectional study, we investigated potential factors associated with SARS-CoV-2 IgG titer levels in Chinese whole-blood donors who have recovered from confirmed or suspected COVID-19 infection.

Our analysis of 386 blood donors revealed a positive correlation between increasing BMI and high-titer IgG levels in CCP among male donors. However, individuals who exercised 1 to 2 times per week or who smoked were less likely to have high-titer IgG levels. Existing research shows that exercise has been identified as a behavioral factor that can enhance immune function in some cases and therefore may act as an adjuvant for immune response. The continuity of exercise intensity and duration may contain different effects on immune function: chronic exercise or training, or high levels of physical activity over a long period of time (months/years), and acute exercise alone: a single exercise session (minutes/hours). (PMID: 24126151) The “J-shaped” association between exercise and infection had the best protection at moderate activity levels (PMID: 21041243) (PMID: 32454138) (PMID: 27535991). According to the American Heart Association's 2022 guidelines for Cardiovascular Health Assessment, Life's Essential 8 mentions that the optimal level is 150 min or more of moderate physical activity per week, or 75 min of vigorous physical activity per week for adults, which means walking for 30 min five times per week. Or running about 25 min three times a week is enough to meet this standard. It is clear that people who exercise almost every day can easily meet this standard, while exercising 1–2 or 3–4 times a week may not be enough to achieve a moderate amount of exercise and lead to a very different effect on immune function. Numerous studies have examined the association between BMI and immune response, with varying results^14,19^. A Colombian study showed a significantly positive association between BMI and IgG levels after COVID-19 infection (*p* < 0.001)^19^. Similarly, another study in the United States reported a positive association between BMI and mean IgG level and neutralizing titers (*p*-value = 0.0008 and 0.0018, respectively)^14^. Some studies have investigated the association between exercises and low-titer IgG values in CCP^16,20^. In our study, blood donors who exercised 1 to 2 times per week were less likely to have high titer IgG levels than those who did not exercise or performed little exercise. Previous studies have indicated that elevated levels of IgG may serve as an indicator of an underlying inflammatory state^21,22^.

Numerous studies evaluated the impact of tobacco smoking and its susceptibility to COVID-19. At present the findings of these studies remain controversial. Some studies have shown that smoking might increase the risk of respiratory tract infections^23,24^. However, other studies found no association between smoking and the severity of Covid-19^15,25^. In our study, we observed that smokers were more likely to have lower IgG titer values. It has been demonstrated that nicotine exerts detrimental effects on B-cell development and IgG production by stimulating immunosuppressive hormones and the nicotinic acetylcholine receptor alpha-7 subunit, which inhibits T and B lymphocyte expression^26,27^.

We also performed an additional subgroup analysis based on gender. Our findings indicated that BMI, smoking, and exercise had an impact on the IgG titer values only in male COVID-19 convalescent blood donors. Several studies have reported on the gender-specific differences in the immune response to COVID-19 infection and vaccines^28–30^. Studies have shown that alcohol intake can lower the SARS-CoV-2 IgG titer levels in COVID-19 convalescent blood donor patients^31,32^. However, in our study, we found no association between alcohol consumption and IgG titer levels. This finding may be attributed to variations in the dose–response relationship associated with each level of alcohol consumption, temporal attenuation of antibodies, and disparities in genetic mutations within alcohol-metabolizing enzymes across different racial groups^33^. At the same time, it may have something to do with our limited sample size.

Our study has several limitations that have to be acknowledged. We only assessed a subset of parameters that could influence the IgG level in convalescent COVID-19 patients. However, several other factors could have affected the IgG titer levels in our population, including the virus strain causing the infection, the patient’s vaccination status. Since our dataset was relatively small and collected from a single center at a specific point in time, the generalizability of our research findings may be restricted. Furthermore, the selective collection of specific blood types, influenced by changing demand over time, may have introduced a bias. Consequently, our findings may not accurately represent the true IgG levels across the entire Chinese population. Therefore, it is essential to conduct larger, multicenter, and longitudinal studies to gain a more comprehensive understanding of how IgG levels change within the Chinese CCP over time and to validate our findings.

## Materials and methods

### Ethics approval and consent to participate

The study was conducted in accordance with the Declaration of Helsinki and granted approval by the Ethics Committee of Chengdu Blood Center. All methods were performed in accordance with relevant guidelines and regulations. Written informed consent was obtained from every enrolled donors or their guardians.

### Population

**Figure 1:**
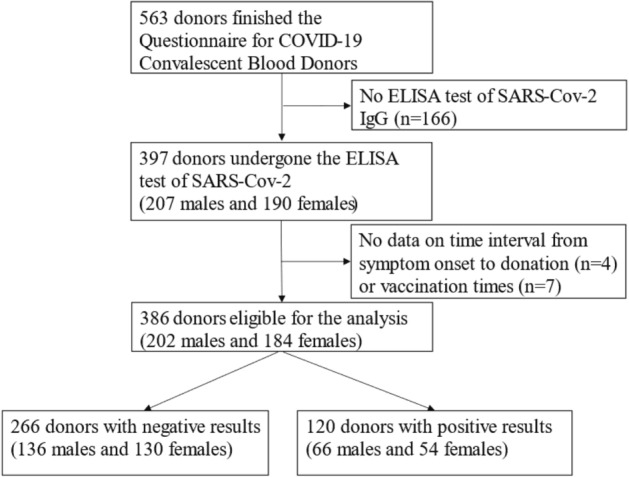
Flow chart.

The study was conducted in Chengdu from December 2022 to March 2023, when the Chinese government adjusted its pandemic prevention strategy and lifted the majority of social travel restrictions. All donors satisfied the standard eligibility criteria for blood donation ruled by the 2019 Technical Operating Regulation for Blood Station^34^ (for example, no history or high-risk factors of transfusion-transmitted infections such as HIV, hepatitis B virus, hepatitis C virus, and Treponema pallidum). Donors who had confirmed or suspected SARS-CoV-2 infection underwent screening, and whole blood was collected at least 7 days after their most recent positive nucleic acid or antigen test result. A total of 563 donors completed the COVID-19 Convalescent Blood Donors Questionnaire, with 397 donors undergoing ELISA testing for SARS-CoV-2 (207 males and 190 females). However, data on the time interval from symptom onset to donation (n = 4) or vaccination time (n = 7) was not available. Ultimately, a total of 386 eligible donors were included in the analysis (202 males and 184 females) (Fig. 1).

### Measurements of other variables

The basic demographic information, including weight, height, age, and sex information was extracted from the Blood Donor Registration. Form which was finished by all eligible blood donors. Information on lifestyle was extracted from Questionnaire for COVID-19 Convalescent Blood Donors a self-reported questionnaire. The alcohol consumption, smoking frequency, exercise frequency, and time duration of sports/exercises information was determined according to participants’ responses to the following question:

If not otherwise specified, please answer the following questions according to the situation in the most recent month:

1. Your alcohol consumption is: (1) Never (2) Quited (3) Currently drinking, ≤ 2 times/week (4) Currently drinking, 3-4 times/week (5) Almost every day
2. Your smoking status is: (1) Never (2) Quited (3) Currently smoking with no more than 19 cigarettes per day (4) Currently smoking with 20 or more cigarettes per day
3. Your sport/exercise frequency before the disease was: (1) hardly any (2) 1-2 times/week (3) 3-4 times/week (4) almost every day

Your sport/exercise time duration is: (1) less than 2 h/week (2) 3–4 h/week (3) 5 h or more/week.

The timing of a blood donor's last vaccination was determined based on the participants' responses to the following questions in the questionnaire: Have you been vaccinated against COVID-19? (If you have been vaccinated, please fill in the number of vaccinations and the last vaccination time).

### Measurements of SARS-CoV-2 IgG antibody titer

The blood samples were diluted 160-fold and tested using the WANTAI SARS-CoV-2 IgG ELISA (Quantitative) (Wantai Biological Pharmacy Enterprise Co, Beijing) via an ML-FAME 24/30 fully automated enzyme immunoassay analyzer (Hamilton Bonaduz AG, Switzerland) as previously reported^35^.

### Statistical analysis

The CCP participants were classified as having high titer SARS-CoV-2 IgG if their values were exceeded or equal to 1:160. The continuous variables were presented as means ± standard deviations (SDs), while the categorical variables were presented as proportions. Logistic regression models were used to evaluate the sex-specific odds ratio and the 95% confidence interval (OR 95% CI) of the different variables on the SARS-CoV-2 IgG titer levels. We further conducted multivariate Logistic regression analyses adjusting for age, times of vaccination, diagnosis (confirmed or suspected COVID-19 infection), and time interval (within 4 weeks or after 4 weeks). Overweight is adjusted further for alcohol intake, smoking status, frequency and time duration of sports/exercises. Other variables were adjusted further for each other variable. The last model was adjusted further for time interval from last vaccination to blood donation (quartiles), and the missing data for this variable (n = 83) was included as a group of the categorical variable in the model.The Statistical Analysis System (SAS) software version 9.4 was used for all statistical analyses and a two-sided *p*-value below 0.05 was deemed statistically significant.

### Conclusion

BMI, smoking habits, and exercise frequency appear to be predictive factors for IgG levels in convalescent male blood donors. However, these variables were not observed as predictive of IgG levels in female convalescent blood donors.

## Data Availability

The databases used and analyzed during the current study are available from the corresponding author on reasonable request.
